# Simultaneous observation of the quantization and the interference pattern of a plasmonic near-field

**DOI:** 10.1038/ncomms7407

**Published:** 2015-03-02

**Authors:** L Piazza, T.T.A. Lummen, E Quiñonez, Y Murooka, B.W. Reed, B Barwick, F Carbone

**Affiliations:** 1 Laboratory for Ultrafast Microscopy and Electron Scattering, ICMP, École Polytechnique Fédérale de Lausanne, Station 6, CH-1015 Lausanne, Switzerland; 2 Department of Physics, Trinity College, 300 Summit Street, Hartford, Connecticut 06106, USA; 3 Physical and Life Sciences Directorate, Lawrence Livermore National Laboratory, 7000 East Avenue L-356, Livermore, California 94551, USA

## Abstract

Surface plasmon polaritons can confine electromagnetic fields in subwavelength spaces and are of interest for photonics, optical data storage devices and biosensing applications. In analogy to photons, they exhibit wave–particle duality, whose different aspects have recently been observed in separate tailored experiments. Here we demonstrate the ability of ultrafast transmission electron microscopy to simultaneously image both the spatial interference and the quantization of such confined plasmonic fields. Our experiments are accomplished by spatiotemporally overlapping electron and light pulses on a single nanowire suspended on a graphene film. The resulting energy exchange between single electrons and the quanta of the photoinduced near-field is imaged synchronously with its spatial interference pattern. This methodology enables the control and visualization of plasmonic fields at the nanoscale, providing a promising tool for understanding the fundamental properties of confined electromagnetic fields and the development of advanced photonic circuits.

## Abstract (web summary)

Mapping the field of surface plasmon polaritons is important to understand their fundamental properties and behaviour. Here, the authors show that ultrafast transmission electron microscopy can simultaneously obtain the spatial interference and quantization of nanowire-confined plasmonic fields.

## Graphical abstract

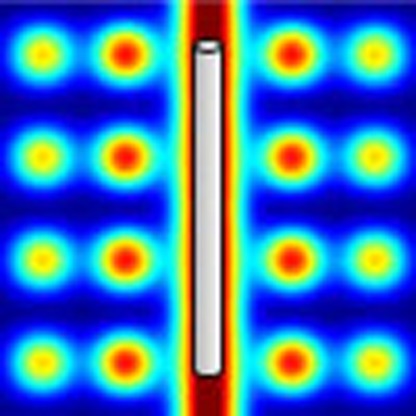

Far-field electromagnetic radiation can be converted to localized electromagnetic energy through the excitation of so-called surface plasmon polaritons (SPPs) at the interface between a metal and a dielectric. The definition refers to the coupling between collective oscillations of electrons in the metal, that is, surface plasmons, and the resulting radiated electromagnetic field, the polariton, which is evanescently confined in the direction perpendicular to the interface. The term polariton is used to define a field that is strongly coupled to a dipolar excitation, which in the case of SPPs is provided by the distribution of electrons in the metal^1^. While SPPs are bound to the metal surface in the perpendicular direction, they can propagate non-radiatively along the surface, and as such can be guided relatively unperturbed through bends, corners or virtually any arbitrary metallic nanostructure^2^,^3^,^4^,^5^. Moreover, SPPs have been shown to be very sensitive to the refractive index of the local dielectric environment, allowing their properties to be tailored using applied electric or magnetic fields in composite nanomaterials systems.^6^ In addition, in materials that exhibit exotic electronic properties due to dimensional confinement, SPPs exhibit unique features such as low-loss propagation and an unusually high modal index^7^,^8^,^9^. Such phenomena are widely investigated because of their potential application in nanophotonic circuits, where subwavelength guiding of the electromagnetic field is crucial to miniaturization^10^,^11^.

For these reasons, a great deal of attention is currently devoted to the observation of SPPs at the nanoscale. Optical near-field techniques can be used to image evanescent fields on surfaces reaching subwavelength spatial resolution in the most advanced set-ups^12^,^13^,^14^, but involve significant drawbacks in terms of signal intensity, the necessity of point-by-point acquisitions and the potential influence of the scanning tip on the electromagnetic near-field being probed. In electron microscopy, imaging through electron energy loss spectroscopy (EELS) has proven successful in mapping SPPs confined by nanostructures with nm resolution^15^,^16^,^17^, and recently Rossouw and Botton^5^,^18^ imaged electron-excited, Fabry–Pérot (FP)-type SPP standing waves in isolated nanowires using a combined scanning transmission electron microscopy (STEM)-EELS approach.

Alternatively, photoexcitation and subsequent EELS imaging of SPPs using the time-resolved photon-induced near-field electron microscopy (PINEM) technique has recently demonstrated additional control of the SPP properties, as well as the possibility to film their evolution in the femtosecond (fs) time domain^19^,^20^. These experiments allow the observation of SPPs in multiple dimensions; space, energy and time, yielding unprecedented insight into their fundamental properties.

In the present work, an SPP standing wave is photoinduced on an isolated metallic nanowire using an intense fs laser pulse, and the ability to control its spatial interference pattern is demonstrated by tuning the polarization of the excitation light. A snapshot of the interaction between the imaging electrons and the SPP standing wave is taken using a new ultrafast-imaging methodology that utilizes an electron imaging filter^21^ to form a two-dimensional (2D) projection of one spatial coordinate versus electron energy. These energy-space-resolved images simultaneously yield both the quantization of the photoinduced SPP field and its characteristic FP interference pattern, given by the one-dimensional (1D) confinement of the single nanowire.

## Results

### SPP imaging

When irradiated by light, isolated metallic nanowires have been shown to behave as quasi-1D plasmonic nanoantennas, whose radiation patterns are governed by the properties of the excitation (wavelength, incident and polarization angles) and their geometry with respect to the orientation and aspect ratio of the wire^22^,^23^. The incident light photoexcites propagating SPPs in the metallic nanowire whose back-and-forth reflections from the wire ends give rise to an SPP standing wave, making the straight nanowire the plasmonic equivalent of an FP nanoresonator^14^. In general, the confined electromagnetic fields of such SPP standing waves, whether electron- or photoinduced, are captured through scanning-based techniques such as combined STEM-EELS^5^,^18^,^24^ or scanning near-field optical microscopy (SNOM)^25^, which probe the electric near-field component perpendicular to the sample plane. Here we alternatively employ a fundamentally different, field-of-view approach, based on ultrafast transmission electron microscopy (UTEM).

**Figure 1:**
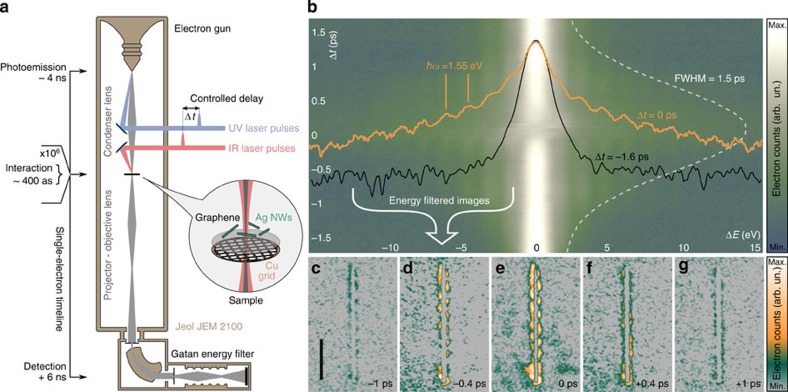
PINEM on a single nanowire. (**a**) A schematic of the experimental set-up. Light and electron pulses at a variable time delay are spatially overlapped on an isolated Ag nanowire suspended on a TEM grid with a few-layer graphene support layer. Probing electrons are detected using a CCD camera after passing through an electron imaging filter. (**b**) Map of the electron energy loss intensity versus the relative time delay Δ*t* between the optical pump and electron probe pulses, taken on a single photoexcited nanowire (5.7 μm length, ≃67 nm radius). Excitation wavelength and polarization angle are 800 nm and *ϕ*=45°, respectively. Energy spectra at negative (Δ*t*=−1.6 ps, black trace) and zero delay (Δ*t*=0 ps, orange trace) are superimposed. The intensity in both the map and the spectra is plotted on a logarithmic scale. (**c**–**g**) Snapshots of an isolated nanowire at different time delays obtained using only the electrons that have gained energy, that is, those in the region indicated by the white arrow in **b**. Electron counts are on a linear scale. The vertical scale bar in **c** corresponds to 2 μm and holds for all images.

UTEM is typically performed by modifying a conventional TEM such that ultrashort bunches of imaging electrons, containing at most one particle each^26^,^27^, can be photoemitted from the cathode by fs laser pulses. Optical access is also provided for photoexcitation of the specimen, and the delay between the two pulse trains is controlled via an optical delay line, allowing for time-resolved optical-pump/electron probe experiments (see Fig. 1a and Methods)^27^,^28^. When the specimen being imaged is a (metallic) nanostructure, the temporal and spatial evolution of a photoinduced SPP standing wave can be visualized via the PINEM imaging technique^19^,^20^. PINEM relies on the inelastic exchange of energy quanta between the photoinduced electromagnetic SPP wave and the relativistic imaging electrons, which probe the SPP electric field component along the electron propagation direction^20^,^29^,^30^,^31^,^32^,^33^. In our experiments, Ag nanowires (~50 nm radius, few-μm length) are isolated and dispersed on a graphene-covered TEM grid and photoexcited using a pulsed 800-nm laser beam at a 5-mJ cm^−2^ fluence, corresponding to a peak excitation energy density of ≃10 GW cm^−2^. Under these experimental conditions, excitation of SPPs by the electron probe beam is a much weaker effect, which can be considered entirely negligible^32^. The few-layer graphene substrate is used to efficiently dissipate the laser-induced heat.

Figure 1b shows the energy spectra of the probing electrons before and after interaction with an isolated, photoexcited Ag nanowire. The spectrum at negative delay (Δ*t*=−1.6 ps) shows the initial energy distribution of the electron bunches, that is, the zero-loss peak (ZLP), whose full-width half-maximum (FWHM) determines the spectral electron energy resolution as better than 1.1 eV. By contrast, when the optical pump and electron probe are overlapped (Δ*t*=0 ps), the interaction with the photoinduced SPP electric field leads to acceleration (deceleration) of the probing electrons, and the corresponding quantized gain (loss) of energy (peaks found at Δ*E*=±*n* · *ℏω*). In the resulting electron energy spectrum, the (net) exchange of up to nine energy quanta can be observed. The panel further depicts the continuous temporal evolution of this electron energy spectrum as a function of Δ*t*, showing a 1.5-ps FWHM cross-correlation of the optical pump and electron probe pulses. As was previously shown, when optimally configured for time resolution, the same system can readily achieve a sub-ps temporal cross-correlation^27^.

The electromagnetic field of the photoexcited SPP in the nanowire can be captured by using an imaging energy filter to select out only electrons that have gained energy (see white arrow in Fig. 1b), and subsequently reforming an image. Repeating this procedure at different time delays produces a series of images (Fig. 1c–g) of the temporal evolution of the SPP field, showing its interferometric standing wave pattern in the silver FP nanoresonator. This type of modal interference pattern reveals the wave character of the electromagnetic SPP field, and is typically observed when the properties of light excitation (wavelength and polarization) are close to a resonance condition of the excited nanowire.

### Plasmonic nanoresonators

**Figure 2:**
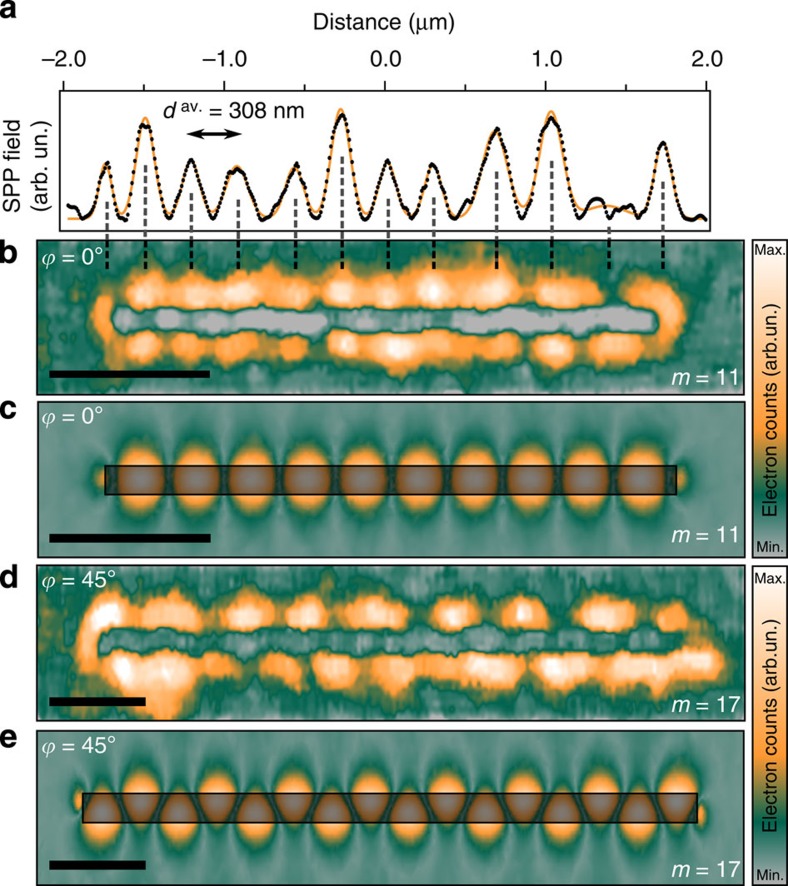
Control of the surface plasmon-polariton field. (**a**) Spatial variation of the interferometric SPP field along the axis of the nanowire imaged in **b**. Black data points depict the background-subtracted SPP field strength integrated along the transverse direction, with the average distance between antinodes *d*^av.^ determined from a multi-Gaussian fit (solid line). (**b**) Experimental PINEM image of the photoinduced SPP field distribution on an isolated nanowire (3.4 μm length, ≃45 nm radius) with light excitation polarized parallel to its longitudinal axis (800 nm, *ϕ*=0°). The image was recorded at Δ*t*=0 ps, using only electrons that have gained energy. Electron counts in **b**–**e** are plotted using the same linear colour scale. The scale bar corresponds to 1 μm. (**c**) Corresponding finite-element simulation of the SPP field (|*E*_z_| in the plane 10 nm below the wire) in the 800 nm, *ϕ*=0° geometry. The shaded area indicates the spatial projection of the nanowire, and the scale bar corresponds to 1 μm. (**d**) Experimental PINEM image of the SPP field distribution (at Δ*t*=0 ps, using only electrons that have gained energy) on an isolated nanowire (5.7 μm length, ≃67 nm radius) under 800 nm, *ϕ*=45° excitation. The scale bar corresponds to 1 μm. Different wires were used for the two polarizations. (**e**) Corresponding finite-element simulation of the SPP field (|*E*_z_| in the plane 10 nm below the wire) in the 800 nm, *ϕ*=45° geometry. The shaded area indicates the spatial projection of the nanowire, and the scale bar corresponds to 1 μm.

For symmetry reasons, light at normal incidence that is polarized parallel to the wire long axis exclusively excites odd-order SPP modes, that is, modes that have an odd number of SPP field nodes *m* (ref. 14). By contrast, electron-excitation of SPPs involves no such symmetry-based selection rules, such that in STEM-EELS both odd- and even-order SPP modes can be excited. To be able to photoexcite even-order SPP modes as well, one requires an excitation geometry where the light is incident at an oblique angle and the azimuthal angle between the light polarization and the wire long axis is nonzero^14^. In general, under such photoexcitation conditions (in *s*-configuration), different SPP modes can be excited at the same time. This can result in non-trivial photoinduced SPP field distributions, which require numerical simulations to reproduce and understand them^13^. As shown in Fig. 2, these general selection rules are valid for the photoexcitation of SPPs in PINEM as well. First, we image a 3.4 μm long, ≃45 nm radius nanowire, illuminated by *s*-polarized light with an azimuthal angle of *ϕ*=0° with respect to the wire long axis, see Fig. 2a,b. The resulting photoinduced SPP standing wave corresponds to an odd-order mode (*m*=11), in excellent agreement with preceding reports^13^,^14^,^23^, and with our own finite-element simulation (see Methods) shown in Fig. 2c. The experimentally estimated SPP wavelength (
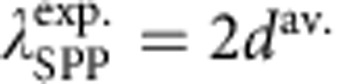
) and wavevector (
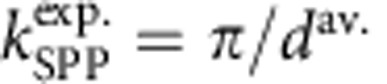
) for this mode are ≃615 nm and ≃10.2 μm^−1^, respectively, from the average antinode distance *d*^av^≃308 nm in the standing wave pattern (see the spatial profile in Fig. 2a). At an energy of 1.55 eV, this is in excellent agreement with both the calculated and experimental dispersion curves of resonant SPP waves in silver nanoantennae^5^,^13^,^18^,^34^. In general, the resonance condition of an order-*m* SPP mode in a 1D FP resonator of length *L* can be written as^14^,^18^:




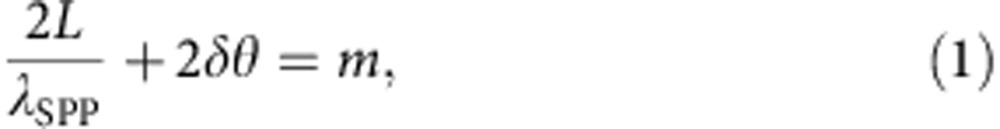




where *δθ* is the SPP phase shift on reflection from the resonator ends, which is often negligible for higher-order modes^13^,^18^. Calculating the expected SPP wavelength for a resonant mode of order *m*=11 in an idealized 1D FP resonator of length *L*=3.4 μm (*δθ*=0) yields a value of 618.2 nm, which is in good agreement with the estimated 
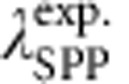
. The slight difference, disregarding the estimation error margin, would imply a negative reflection phase shift *δθ* according to equation (1). However, it is more intuitively interpreted in terms of a shortened effective wire length *L*^eff.^, resulting from the fact that the hemispherical wire caps were included in the determination of *L*^14^,^18^.

Next we illuminate a 5.7 μm long nanowire using light polarized at a *ϕ*=45° azimuthal angle, obtaining a non-trivial SPP field distribution in which antinodes of opposite phase are concentrated on alternating sides of the wire (Fig. 2d). Under these excitation conditions, the nodal lines are clearly at an angle with respect to the long axis of the nanowire. As shown in Fig. 2e, the features of this transversally asymmetric SPP mode (*m*=17) are numerically accounted for by our finite-element simulations. The continuous dependence of the simulated SPP field distribution on the azimuthal polarization angle of the light excitation is illustrated in Supplementary Movie 1. To our knowledge, the experimental observation of such an asymmetric distribution of the SPP field using either SNOM or STEM-EELS techniques has never been reported. In principle, these features could also be observed in SNOM experiments^14^. The absence of such asymmetric field distributions in STEM-EELS experiments is likely related to the differences between electron- and photoexcitation of SPPs in terms of mechanism and selection rules^20^,^24^,^31^,^32^,^33^. For photoexcited SPP modes, Dorfmüller *et al*.^14^,^23^ showed that while odd-order modes emit the strongest field in the direction perpendicular to the nanowire, thus maximizing the PINEM effect, the even-order modes have a minimum of radiation in this direction, resulting in a more difficult detection. However, at the same time, the relative dipole coupling strength of odd-order modes was shown to quickly decrease with *m*, while staying fairly constant for even-order modes. In our experiments, two different nanowires were used to maximize the strength of the photoinduced SPP field in the different excitation geometries. The excellent agreement between the experimental and simulated SPP field distributions demonstrates the potential of controlling SPPs using an external light field.

### Energy-space SPP mapping

**Figure 3:**
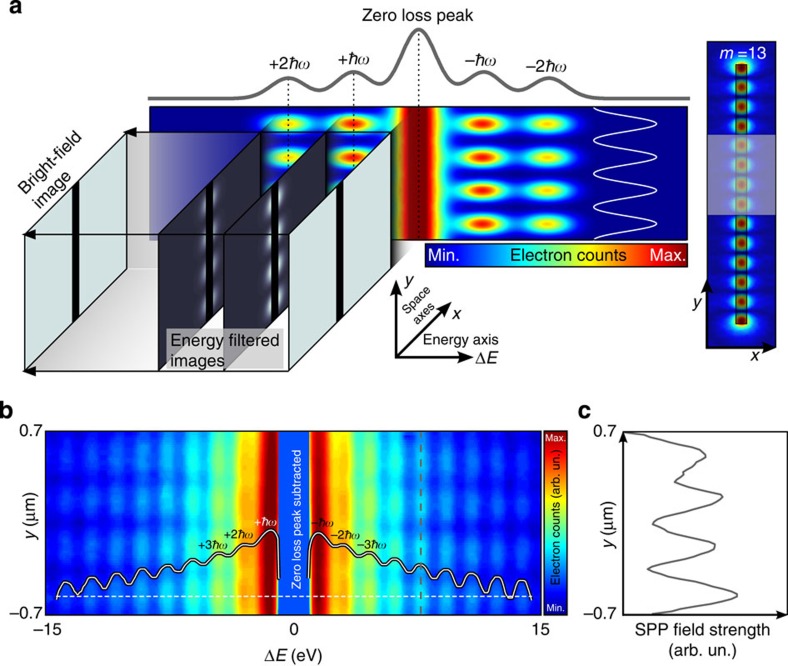
Energy-space imaging. (**a**) Conceptual representation of the energy-space-resolved PINEM methodology. Rather than recording an energy-filtered 2D spatial map of the transmitted electrons (images on the left), or dispersing the electrons only in energy (spectrum on top), this method retains the spatial electron distribution along the vertical axis, while also dispersing the electrons according to their energy along the horizontal axis. Combined with the PINEM effect, this results in the vertical spatial variation of the photoinduced SPP field being duplicated at equidistantly spaced energy quanta, with an intensity envelope and energy resolution determined by the PINEM interaction strength and the ZLP-width, respectively. The vertical spatial variation here (solid white trace) corresponds to a selected part (white shaded area) of the simulated photoinduced field (|*E*_z_| in the plane 10 nm below the wire) of an isolated nanowire (black shaded rectangle, 4.6 μm length, ≃61 nm radius, 800 nm excitation, *ϕ* = 0°, *m*=13), indicated on the right. Electron counts in both images are plotted on the same linear scale. (**b**) The experimentally obtained energy-space image, taken on a selected section of a photoexcited nanowire (4.6 μm length, ≃61 nm radius, 800 nm excitation, *ϕ* = 0°, Δ*t*=0 ps) is displayed together with a horizontal cut (along the energy axis, white dashed line), showing the quantized energy dependence of the interferometric spatial distribution of the SPP field. A Gaussian-fitted ZLP peak was subtracted and the intensity (electron counts) is mapped on a logarithmic scale in both the image and the spectrum to enhance the contrast. (**c**) A vertical cross-section at the energy corresponding to the net exchange of five photon quanta (grey dashed line in **b**) is shown, depicting the spatial distribution of the plasmonic field with its characteristic interference fringes.

After interacting with the photoinduced SPP field, the imaging electrons carry all the information about the exchange encoded in their spatial and energy distributions. In EELS and 2D energy-filtered imaging, one typically collapses one or more of these coordinates to obtain either a 1D energy spectrum (collapsing both spatial coordinates) or a 2D image (collapsing the electron energy). Instead, to simultaneously observe both the quantized spectrum and its spatial distribution, here we resonantly excite an odd-order SPP mode in an isolated nanowire and align the corresponding image such that the wire long axis is parallel with the vertical detector axis (see Fig. 3a). By then collapsing only the perpendicular (horizontal) spatial coordinate, we obtain an image that contains spectroscopic information along the horizontal detector axis and spatial information along the vertical detector axis. The experimental energy-space map is shown in Fig. 3b. To optimally resolve the inelastic exchange process, we zoomed in on a selected section of the nanowire (4.6 μm length, ≃61 nm radius, *ϕ* =0°). As is clear from the experimental image, taking a horizontal cut (horizontal dashed line) yields the quantized spectrum of the interaction between the SPP field and the imaging electrons. At the same time, by taking a vertical cut at an energy corresponding to one of the peaks in the energy spectrum (vertical dashed line), the spatial distribution of the interaction between single electrons and a discrete number of photons is obtained, Fig. 3c, displaying the typical interference fringes of the resonant SPP standing wave. Though both the wave and particle character of SPPs were already observed separately in individual, tailored experiments^35^,^36^,^37^, here we obtain a very direct and illustrative view of both aspects of the SPP field simultaneously in a single experiment.

## Discussion

It is important to clarify that the PINEM energy-space map shown in Fig. 3b is fundamentally different from those previously obtained in STEM-EELS experiments^5^,^18^,^24^,^38^. In the latter, the transient field of the fast electron probe excites all the different SPP modes across a wide energy range. Accordingly, the multiple peaks observed in the electron energy distribution correspond to the different odd- and even-order SPP resonances of the excited nanoresonator, and energy-filtered imaging centred on these different SPP resonances visualizes their correspondingly different spatial field distributions. By contrast, in PINEM, the SPP modes are excited by light with a fixed wavelength, which selectively drives only the SPP modes at the corresponding energy, and even then only when they are symmetry matched to the excitation geometry. Any optically driven SPP mode thus has an energy equal to that of the incident photons, which is retrieved as the energy spacing of the series of equidistant peaks in the electron energy gain/loss spectrum of a PINEM experiment (see Fig. 1b). Rather than being related to the different eigenmodes of the FP nanoresonator, these peaks correspond to the exchange of different discrete numbers of SPP field quanta with the probing electrons. As a consequence, regardless of the relative electron energy at which one images, the observed SPP field distribution corresponds to the optically driven mode (see Fig. 3b).

**Figure 4:**
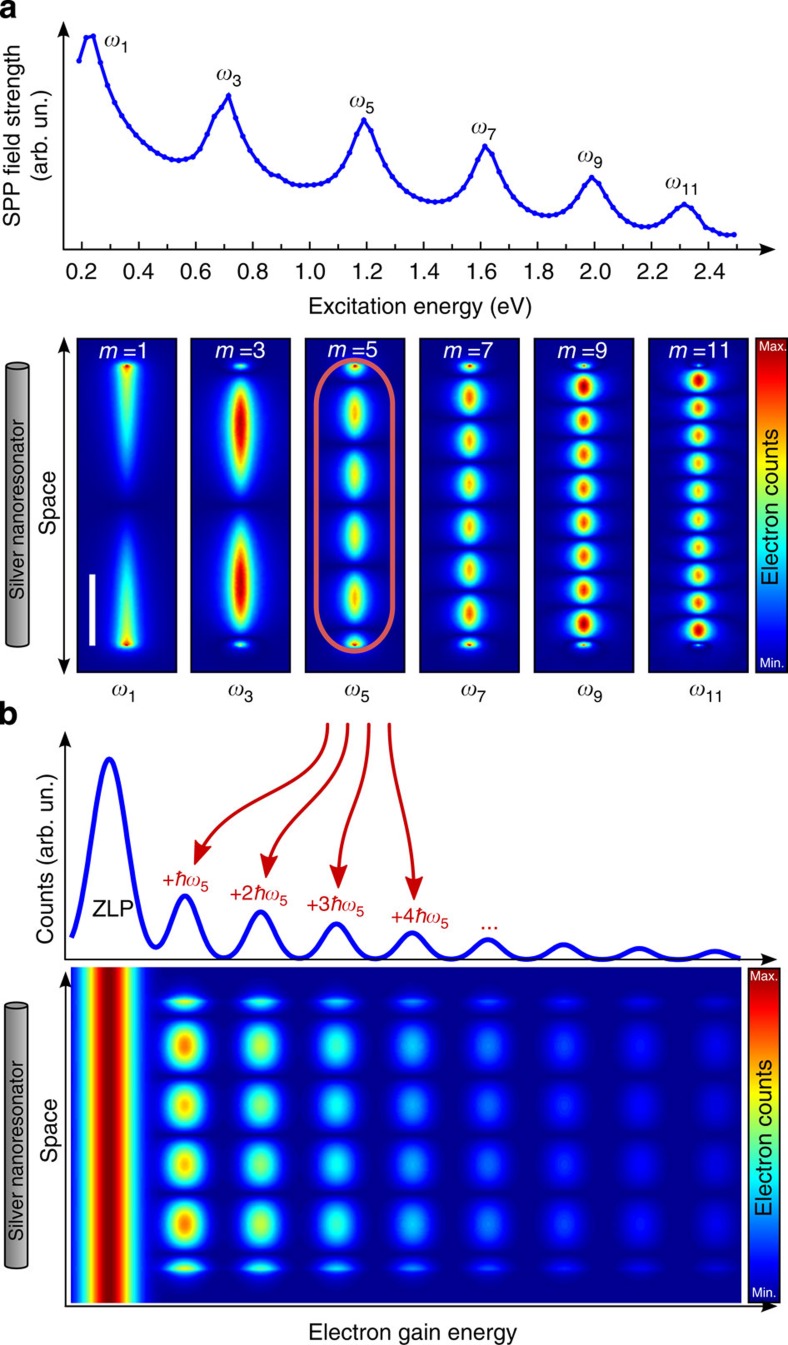
Excitation energy-dependent imaging versus energy-space imaging. (**a**) Finite-element simulation of the excitation energy dependence of the photoinduced SPP field strength (log_10_ |*E*_z_| integrated over the volume surrounding the nanowire) in a *ϕ*=0° normal incidence excitation geometry. The simulated nanowire length and radius are 2 μm and 40 nm, respectively. Representative spatial field distributions of the various odd-order SPP resonances are shown below (|*E*_z_| in the plane 10 nm below the wire), with electron counts plotted using the same linear colour scale. The vertical scale bar in the image of the *m*=1 SPP mode corresponds to 500 nm and holds for all images. (**b**) Selectively photoexciting only one of the SPP modes of this wire (here *m*=5) in a single-wavelength PINEM experiment instead (at *ω*_5_, *ϕ*=0°) quantizes the energy exchange of its field distribution with the probing electrons, as shown in this conceptual PINEM energy-space map. The corresponding electron energy gain spectrum is depicted on the top. Electron counts in both the map and the spectrum are plotted on a linear scale. Though not shown here, a similar series of quantized features is present on the energy loss side of the ZLP in both the energy-space map and the spectrum.

To explore the different SPP modes of the nanoresonator using PINEM, one would perform a series of PINEM imaging experiments while varying the wavelength of the light excitation. The simulation in Fig. 4a, carried out for light polarized parallel to the wire long axis (2 μm length, 40 nm radius), illustrates the typical wavelength dependence of the strength of the photoinduced SPP field, and highlights the resonant SPP field distributions. The continuous wavelength dependence of the photoinduced field distribution can be seen in Supplementary Movie 2. In agreement with previous experiments and simulations^13^,^14^,^23^, only odd-order SPP modes are photoexcited in this *ϕ*=0° excitation geometry. A PINEM experiment at a single wavelength allows one to selectively photoexcite a single SPP mode (circled in Fig. 4a), which then exchanges energy quanta corresponding to that free space wavelength with the probing electrons, yielding the equidistantly peaked electron energy spectrum shown in Fig. 4b. Aligning a nanoresonator supporting such a single photoexcited SPP mode along the vertical detector axis, and simultaneously projecting out the spatial and energy distribution of the probing electrons along the vertical and horizontal detector axes, respectively, yields the energy-space map that showcases the wave–particle duality of the SPP (see Figs 3b and 4b).

Owing to the strong optical excitation in PINEM, even when the excitation wavelength falls between the SPP resonant conditions of a nanoresonator, the inelastic exchange of energy quanta can be observed. Corresponding energy-filtered PINEM images can thus provide the spatial distribution of the optically driven SPP near-field away from a FP resonance. This approach is in principle only limited by the optical damage threshold of the nanoresonator. The additional control offered by the tunability of the laser excitation intensity enables the possibility to enhance these off-resonance excitations, providing high contrast and high-resolution images of their spatial field distribution. Another advantage of using PINEM to image plasmonic field distributions, in contrast to scanning-based conventional STEM-EELS imaging, is the capability of capturing the dynamics of the photoinduced SPP fields with fs resolution^19^,^20^.

Summarizing, in ultrafast energy-filtered PINEM imaging, each pulse contains at most one electron to avoid space-charge broadening, and the electrons that exchanged quanta with the photoinduced SPP field carry the information about the interaction between one single electron and a discrete number of exchanged photons. An intriguing consequence is that by imaging the energy spectrum of such an exchange as well as its spatial distribution at the same time, one can obtain simultaneous information on complementary aspects of the confined electromagnetic field^39^,^40^,^41^. The wave aspect of SSPs was previously established in an SPP analogue to Young’s famous double-slit experiment^35^. Furthermore, SPPs have also been shown to be quantized in the same way as the free space electromagnetic field (that is, light), manifesting similar quantum behaviour including entanglement^42^ and two-plasmon quantum interference^37^,^43^. Recently, single SSPs were shown to exhibit both wave and particle behaviour, in a set of individual measurements^36^. Our experiments provide a simultaneous observation of these two aspects of the SPP field in a single measurement and demonstrate the ability of a UTEM to image and control SPPs in multiple dimensions of space, energy and time, yielding further insight into their behaviour, and providing a unique playground for the observation of the fundamental properties of confined electromagnetic fields.

## Methods

### Sample preparation

Hemispherically capped silver nanowires (radii ~50 nm, varying lengths on the order of a few-μm)^44^ were dispersed in dimethylformamide (Acros Organics, 99.95% purity) through ultrasonification for 10 min. Samples were prepared by dropcasting a single drop of the resulting suspension on a 300-mesh copper TEM grid covered by a few-layer graphene support film on lacey carbon (Ted Pella, 21740), immediately following ultrasonification. TEM grids were then air dried for a minimum of 4 hours before examination in the UTEM (at 295 K and ~10^−5^ Pa) for the identification and characterization of isolated silver nanowires.

### Experimental apparatus

A 500-kHz train of linearly polarized, 800-nm, 80-fs light pulses was split to generate two beams. One of these beams was frequency tripled to deliver few-nJ ultraviolet pulses that were used to photoemit electrons from the custom truncated-cone LaB_6_ tip (15 μm diameter truncation plane, AP-Tech) of the thermionic electron gun in a modified JEOL JEM 2100 microscope^27^. The other 800-nm pulsed laser beam was passed through an optical delay line and focused on the sample in the UTEM at near-normal incidence such that photoexcitation in the field-of-view of the photoelectron beam was uniform. Corresponding optical fluences ranged between tens of μJ cm^−2^ and 5 mJ cm^−2^. The experimental apparatus is equipped with a post-column Gatan Quantum GIF electron energy loss spectrometer (GIF)^21^, and is pictorially represented in Fig. 1a. A detailed description and characterization of the system can be found elsewhere^27^. For the PINEM experiments described in this work, the UTEM was operated at 200 keV in photoelectron mode. The GIF imaging camera was operated with a dispersion setting of 0.05 eV per channel, and typical exposure times of the 2,048 × 2,048 pixel CCD sensor were 60 s for images and 10 s for spectra. PINEM energy spectra were aligned using a differential-based maximum intensity alignment algorithm, and where appropriate the ZLP was removed by subtraction of a fitted Gaussian line profile.

### Finite-element simulations

The plasmonic near-field around the silver nanowires was calculated using commercial fast finite-element software (COMSOL Multiphysics 4.3b, www.comsol.com), using the Wave Optics package, performing two sequential frequency domain studies. Nanowires were modelled as flat-ended cylinders of varying length and radius, whose complex, wavelength dependent refractive index was taken from Palik^45^. The nanoscatterer was surrounded by a rectangular volume of vacuum (index of refraction taken as 1), which itself was surrounded by perfectly matched layers (layer thickness 150 nm, minimum clearance to nanowire 250 nm) to absorb scattered light and minimize reflections. In a two-step calculation, first the distribution of the excitation electromagnetic field was calculated throughout the physical simulation volume (in the absence of the nanowire). The excitation was modelled as a linearly polarized plane wave incident on the rectangular input port above the scatterer (positive *z*) and absorbed at the corresponding output port below the scatterer (negative *z*). Floquet boundary conditions were imposed on the lateral boundaries of the simulation volume. The resulting electric field was then used as the background field in the second calculation, which solved for the electric field scattered by the nanowire. The maximum mesh element size on the nanowire surface was set to 30 nm, and the meshing of all other domains was chosen to optimize both the computational time and the accuracy of the calculation, similar to the procedure described by Miljković *et al*.^13^ Corresponding simulation details are visually summarized in both Supplementary Movies.

## Additional information

**How to cite this article:** Piazza, L. *et al*. Simultaneous observation of the quantization and the interference pattern of a plasmonic near-field. *Nat. Commun.* 6:6407 doi: 10.1038/ncomms7407 (2015).

## Supplementary Material

Supplementary Movie 1 Simulation of the continuous polarization dependence of the photoinduced SPP field distribution in a silver nanoresonator of 5.7 μm length and 67 nm radius under 800 nm excitation. Details of the simulation model are presented first, followed by the continuous evolution of the induced |*E_z_*| distribution with polarization angle *φ*.

Supplementary Movie 2 Simulation of the continuous excitation energy dependence of the photoinduced SPP field distribution in a silver nanoresonator of 2.0 μm length and 40 nm radius under *φ* = 0° excitation, across an energy range spanning several SPP mode resonances. Details of the simulation model are presented first, followed by the continuous evolution of the induced *E_z_* distribution with excitation energy.
